# *N*′-(2,4-Dimethoxy­benzyl­idene)-3,4,5-trihydroxy­benzohydrazide ethanol solvate

**DOI:** 10.1107/S1600536809018947

**Published:** 2009-05-23

**Authors:** Abeer A. Alhadi, Siti Munirah Saharin, Hapipah Mohd Ali, Ward T. Robinson, Mahmood A. Abdulla

**Affiliations:** a Department of Chemistry, University of Malaya, 50603 Kuala Lumpur, Malaysia; b Department of Molecular Medicine, University of Malaya, 50603 Kuala Lumpur, Malaysia

## Abstract

The title compound, C_16_H_16_N_2_O_6_·C_2_H_5_OH, was synthesized from 3,4,5-trihydroxy­benzoyl­hydrazide and 2,4-dimethoxy­benzaldehyde in ethanol. The compound is not planar, with the two aromatic planes of the two aromatic rings twisted by 15.6 (1)°. The hydr­oxy groups are involved in both intra­molecular O—H⋯O and inter­molecular O—H⋯N and O—H⋯O hydrogen bonds and a C—H⋯O interaction also occurs.

## Related literature

For related compounds, see Abdul Alhadi *et al.* (2009 ▶). For the parent *N*′-(2-hydroxy­benzyl­idene)benzohydrazide, see Lyubchova *et al.* (1995 ▶).
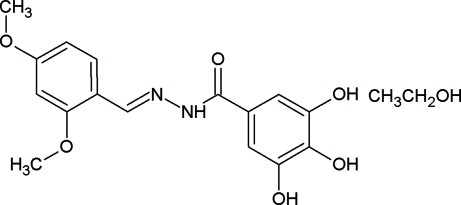


## Experimental

### 

#### Crystal data

C_16_H_16_N_2_O_6_·C_2_H_6_O
*M* *_r_* = 378.38
Monoclinic, 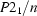

*a* = 7.8347 (1) Å
*b* = 17.5412 (3) Å
*c* = 13.0230 (2) Å
β = 93.936 (1)°
*V* = 1785.53 (5) Å^3^
*Z* = 4
Mo *K*α radiation
μ = 0.11 mm^−1^
*T* = 100 K
0.31 × 0.16 × 0.12 mm

#### Data collection

Bruker APEXII CCD area-detector diffractometer
Absorption correction: multi-scan (*SADABS*; Sheldrick, 1996 ▶) *T*_min_ = 0.967, *T*_max_ = 0.987
19580 measured reflections
5188 independent reflections
3586 reflections with *I* > 2σ(*I*)
*R*_int_ = 0.041

#### Refinement

*R*[*F* ^2^ > 2σ(*F* ^2^)] = 0.044
*wR*(*F* ^2^) = 0.121
*S* = 1.02
5188 reflections
253 parameters
H-atom parameters constrained
Δρ_max_ = 0.41 e Å^−3^
Δρ_min_ = −0.27 e Å^−3^

### 

Data collection: *APEX2* (Bruker, 2007 ▶); cell refinement: *SAINT* (Bruker, 2007 ▶); data reduction: *SAINT*; program(s) used to solve structure: *SHELXS97* (Sheldrick, 2008 ▶); program(s) used to refine structure: *SHELXL97* (Sheldrick, 2008 ▶); molecular graphics: *X-SEED* (Barbour, 2001 ▶); software used to prepare material for publication: *publCIF* (Westrip, 2008 ▶).

## Supplementary Material

Crystal structure: contains datablocks I, global. DOI: 10.1107/S1600536809018947/hg2510sup1.cif

Structure factors: contains datablocks I. DOI: 10.1107/S1600536809018947/hg2510Isup2.hkl

Additional supplementary materials:  crystallographic information; 3D view; checkCIF report

## Figures and Tables

**Table 1:** Hydrogen-bond geometry (Å, °)

*D*—H⋯*A*	*D*—H	H⋯*A*	*D*⋯*A*	*D*—H⋯*A*
O2—H2⋯O3	0.84	2.54	2.9325 (13)	109
O3—H3⋯O2	0.84	2.54	2.9325 (13)	110
O4—H4⋯O3	0.84	2.25	2.7009 (14)	114
N1—H1′⋯O7^i^	0.88	2.04	2.8844 (15)	160
O2—H2⋯O1^ii^	0.84	1.86	2.6871 (13)	170
O2—H2⋯N2^ii^	0.84	2.57	2.9293 (15)	107
O3—H3⋯O1^ii^	0.84	1.88	2.7200 (13)	177
O4—H4⋯O6^iii^	0.84	2.14	2.7366 (14)	127
C14—H14⋯O2^iv^	0.95	2.42	3.3539 (17)	167

Symmetry codes: (i) 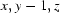; (ii) 
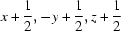
; (iii) 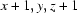; (iv) 
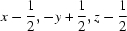
.

**Fig. 1.**
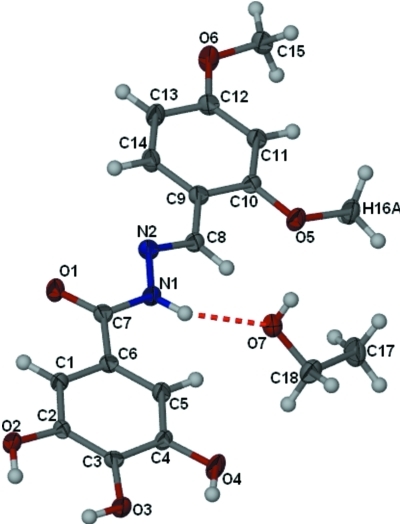
Thermal ellipsoid plot (Barbour, 2001) of C16H16N2O6.C2H5OH at 70% probability level. Hydrogen atoms are drawn as sphere of arbitrary radius.

## supplementary crystallographic information

### Experimental

A mixture of 3,4,5-trihydroxybenzoylhydrazide and 2,4-dimethoxybenzaldehyde were heated in ethanol (50 ml) for 12 h. The yellow crystals were obtained by recrystallization from ethanol.

### Refinement

All H atoms were placed at calculated positions (C— H 0.95 - 0.99, N—H 0.88, and O—H 0.84 Å) with *U*_iso_(H) set to 1.2 - 1.5times *U*_eq_ (C,*N*,*O*)

### Crystal data

**Table:**

C_16_H_16_N_2_O_6_·C_2_H_6_O	*F*(000) = 800
*M**_r_* = 378.38	*D*_x_ = 1.408 Mg m^−^^3^
Monoclinic, *P*2_1_/*n*	Mo *K*α radiation, λ = 0.71073 Å
Hall symbol: -P 2yn	Cell parameters from 3445 reflections
*a* = 7.8347 (1) Å	θ = 2.8–29.1°
*b* = 17.5412 (3) Å	µ = 0.11 mm^−^^1^
*c* = 13.0230 (2) Å	*T* = 100 K
β = 93.936 (1)°	Block, yellow
*V* = 1785.53 (5) Å^3^	0.31 × 0.16 × 0.12 mm
*Z* = 4	

### Data collection

**Table:**

Bruker APEXII CCD area-detector diffractometer	5188 independent reflections
Radiation source: fine-focus sealed tube	3586 reflections with *I* > 2σ(*I*)
graphite	*R*_int_ = 0.041
ω scans	θ_max_ = 30.5°, θ_min_ = 2.0°
Absorption correction: multi-scan (*SADABS*; Sheldrick, 1996)	*h* = −11→10
*T*_min_ = 0.967, *T*_max_ = 0.987	*k* = −24→24
19580 measured reflections	*l* = −18→18

### Refinement

**Table:**

Refinement on *F*^2^	Primary atom site location: structure-invariant direct methods
Least-squares matrix: full	Secondary atom site location: difference Fourier map
*R*[*F*^2^ > 2σ(*F*^2^)] = 0.044	Hydrogen site location: inferred from neighbouring sites
*wR*(*F*^2^) = 0.121	H-atom parameters constrained
*S* = 1.02	*w* = 1/[σ^2^(*F*_o_^2^) + (0.0566*P*)^2^ + 0.3538*P*] where *P* = (*F*_o_^2^ + 2*F*_c_^2^)/3
5188 reflections	(Δ/σ)_max_ = 0.001
253 parameters	Δρ_max_ = 0.41 e Å^−^^3^
0 restraints	Δρ_min_ = −0.26 e Å^−^^3^

### Special details

**Table:**

Geometry. All e.s.d.'s (except the e.s.d. in the dihedral angle between two l.s. planes) are estimated using the full covariance matrix. The cell e.s.d.'s are taken into account individually in the estimation of e.s.d.'s in distances, angles and torsion angles; correlations between e.s.d.'s in cell parameters are only used when they are defined by crystal symmetry. An approximate (isotropic) treatment of cell e.s.d.'s is used for estimating e.s.d.'s involving l.s. planes.
Refinement. Refinement of *F*^2^ against ALL reflections. The weighted *R*-factor *wR* and goodness of fit *S* are based on *F*^2^, conventional *R*-factors *R* are based on *F*, with *F* set to zero for negative *F*^2^. The threshold expression of *F*^2^ > σ(*F*^2^) is used only for calculating *R*-factors(gt) *etc*. and is not relevant to the choice of reflections for refinement. *R*-factors based on *F*^2^ are statistically about twice as large as those based on *F*, and *R*- factors based on ALL data will be even larger.

### Fractional atomic coordinates and isotropic or equivalent isotropic displacement parameters (Å^2^)

**Table:**

	*x*	*y*	*z*	*U*_iso_*/*U*_eq_	
C1	1.15941 (17)	0.21466 (7)	0.76768 (10)	0.0156 (3)	
H1	1.1458	0.2583	0.7248	0.019*	
C2	1.25203 (17)	0.22016 (7)	0.86245 (10)	0.0153 (3)	
C3	1.26837 (17)	0.15678 (8)	0.92716 (10)	0.0154 (3)	
C4	1.19696 (18)	0.08744 (8)	0.89317 (10)	0.0170 (3)	
C5	1.10756 (18)	0.08150 (8)	0.79830 (10)	0.0176 (3)	
H5	1.0605	0.0339	0.7760	0.021*	
C6	1.08672 (17)	0.14571 (7)	0.73529 (10)	0.0154 (3)	
C7	0.98683 (17)	0.14395 (8)	0.63409 (10)	0.0159 (3)	
C8	0.74521 (17)	0.00842 (8)	0.49550 (10)	0.0168 (3)	
H8	0.7539	−0.0296	0.5476	0.023 (4)*	
C9	0.64100 (17)	−0.00596 (8)	0.40063 (10)	0.0165 (3)	
C10	0.57036 (17)	−0.07846 (8)	0.38154 (10)	0.0163 (3)	
C11	0.47202 (17)	−0.09404 (8)	0.29061 (10)	0.0172 (3)	
H11	0.4236	−0.1431	0.2785	0.021*	
C12	0.44633 (18)	−0.03640 (8)	0.21828 (11)	0.0188 (3)	
C13	0.51352 (19)	0.03637 (8)	0.23587 (11)	0.0209 (3)	
H13	0.4939	0.0754	0.1859	0.025*	
C14	0.60860 (18)	0.05093 (8)	0.32640 (11)	0.0190 (3)	
H14	0.6533	0.1007	0.3389	0.023*	
C15	0.2879 (2)	−0.11915 (8)	0.09897 (11)	0.0227 (3)	
H15A	0.3824	−0.1557	0.0978	0.034*	
H15B	0.2270	−0.1169	0.0307	0.034*	
H15C	0.2087	−0.1354	0.1498	0.034*	
C16	0.5321 (2)	−0.20516 (8)	0.44383 (12)	0.0247 (3)	
H16A	0.4072	−0.2007	0.4358	0.037*	
H16B	0.5647	−0.2364	0.5044	0.037*	
H16C	0.5737	−0.2292	0.3824	0.037*	
C17	0.7901 (2)	0.81507 (9)	0.72385 (14)	0.0313 (4)	
H19A	0.6929	0.8370	0.6826	0.047*	
H19B	0.7482	0.7866	0.7818	0.047*	
H19C	0.8531	0.7806	0.6809	0.047*	
C18	0.9073 (2)	0.87827 (9)	0.76416 (12)	0.0259 (3)	
H18A	0.8458	0.9110	0.8113	0.031*	
H18B	1.0074	0.8559	0.8038	0.031*	
N1	0.92094 (15)	0.07647 (6)	0.60343 (9)	0.0168 (2)	
H1'	0.9379	0.0357	0.6422	0.030 (5)*	
N2	0.82556 (15)	0.07168 (6)	0.50974 (9)	0.0174 (2)	
O1	0.96209 (13)	0.20261 (5)	0.58110 (7)	0.0219 (2)	
O2	1.32670 (14)	0.28879 (5)	0.88620 (7)	0.0218 (2)	
H2	1.3569	0.2901	0.9493	0.033*	
O3	1.35316 (13)	0.15568 (6)	1.02261 (7)	0.0209 (2)	
H3	1.3877	0.1998	1.0381	0.031*	
O4	1.21299 (15)	0.02350 (6)	0.95174 (8)	0.0273 (3)	
H4	1.2718	0.0332	1.0065	0.041*	
O5	0.60584 (13)	−0.13118 (6)	0.45673 (7)	0.0224 (2)	
O6	0.35424 (14)	−0.04532 (6)	0.12604 (8)	0.0264 (3)	
O7	0.96498 (14)	0.92367 (6)	0.68270 (8)	0.0229 (2)	
H7	1.0291	0.8975	0.6475	0.034*	

### Atomic displacement parameters (Å^2^)

**Table:**

	*U*^11^	*U*^22^	*U*^33^	*U*^12^	*U*^13^	*U*^23^
C1	0.0190 (6)	0.0138 (6)	0.0137 (6)	0.0025 (5)	−0.0015 (5)	0.0010 (5)
C2	0.0187 (6)	0.0123 (6)	0.0148 (6)	−0.0006 (5)	0.0000 (5)	−0.0015 (5)
C3	0.0175 (6)	0.0163 (6)	0.0119 (6)	0.0006 (5)	−0.0021 (5)	−0.0010 (5)
C4	0.0214 (7)	0.0143 (6)	0.0147 (6)	0.0000 (5)	−0.0029 (5)	0.0027 (5)
C5	0.0223 (7)	0.0140 (6)	0.0159 (7)	−0.0007 (5)	−0.0034 (5)	−0.0003 (5)
C6	0.0171 (6)	0.0156 (6)	0.0131 (6)	0.0011 (5)	−0.0020 (5)	−0.0006 (5)
C7	0.0175 (6)	0.0157 (6)	0.0141 (6)	0.0019 (5)	−0.0016 (5)	−0.0005 (5)
C8	0.0194 (7)	0.0158 (6)	0.0148 (6)	0.0008 (5)	−0.0021 (5)	0.0007 (5)
C9	0.0170 (6)	0.0168 (6)	0.0151 (7)	−0.0002 (5)	−0.0021 (5)	−0.0009 (5)
C10	0.0187 (7)	0.0162 (6)	0.0139 (6)	0.0010 (5)	−0.0010 (5)	0.0013 (5)
C11	0.0188 (7)	0.0162 (6)	0.0161 (6)	−0.0014 (5)	−0.0020 (5)	−0.0015 (5)
C12	0.0201 (7)	0.0192 (7)	0.0162 (7)	0.0000 (5)	−0.0059 (5)	−0.0012 (5)
C13	0.0274 (8)	0.0156 (6)	0.0186 (7)	−0.0001 (6)	−0.0065 (6)	0.0026 (5)
C14	0.0208 (7)	0.0141 (6)	0.0212 (7)	−0.0009 (5)	−0.0040 (5)	−0.0008 (5)
C15	0.0287 (8)	0.0186 (7)	0.0197 (7)	−0.0034 (6)	−0.0074 (6)	−0.0025 (6)
C16	0.0339 (8)	0.0158 (7)	0.0236 (8)	−0.0045 (6)	−0.0042 (6)	0.0033 (6)
C17	0.0369 (9)	0.0197 (7)	0.0381 (10)	−0.0044 (7)	0.0079 (7)	−0.0009 (7)
C18	0.0344 (8)	0.0217 (7)	0.0212 (7)	0.0002 (6)	−0.0020 (6)	0.0049 (6)
N1	0.0215 (6)	0.0151 (5)	0.0129 (5)	−0.0010 (4)	−0.0063 (4)	0.0013 (4)
N2	0.0199 (6)	0.0172 (6)	0.0142 (6)	0.0000 (5)	−0.0064 (4)	−0.0011 (4)
O1	0.0323 (6)	0.0142 (5)	0.0176 (5)	0.0012 (4)	−0.0101 (4)	0.0016 (4)
O2	0.0353 (6)	0.0145 (5)	0.0145 (5)	−0.0057 (4)	−0.0070 (4)	0.0012 (4)
O3	0.0310 (6)	0.0167 (5)	0.0137 (5)	−0.0028 (4)	−0.0079 (4)	0.0011 (4)
O4	0.0446 (7)	0.0167 (5)	0.0184 (5)	−0.0065 (5)	−0.0141 (5)	0.0056 (4)
O5	0.0320 (6)	0.0162 (5)	0.0177 (5)	−0.0042 (4)	−0.0076 (4)	0.0030 (4)
O6	0.0382 (6)	0.0176 (5)	0.0209 (5)	−0.0043 (4)	−0.0160 (5)	0.0010 (4)
O7	0.0295 (6)	0.0163 (5)	0.0232 (6)	0.0001 (4)	0.0034 (4)	−0.0001 (4)

### Geometric parameters (Å, °)

**Table:**

C1—C6	1.3900 (18)	C13—C14	1.3745 (19)
C1—C2	1.3914 (18)	C13—H13	0.9500
C1—H1	0.9500	C14—H14	0.9500
C2—O2	1.3647 (15)	C15—O6	1.4308 (16)
C2—C3	1.3958 (18)	C15—H15A	0.9800
C3—O3	1.3687 (15)	C15—H15B	0.9800
C3—C4	1.3979 (18)	C15—H15C	0.9800
C4—O4	1.3574 (16)	C16—O5	1.4258 (17)
C4—C5	1.3818 (18)	C16—H16A	0.9800
C5—C6	1.3965 (18)	C16—H16B	0.9800
C5—H5	0.9500	C16—H16C	0.9800
C6—C7	1.4862 (18)	C17—C18	1.511 (2)
C7—O1	1.2466 (16)	C17—H19A	0.9800
C7—N1	1.3412 (17)	C17—H19B	0.9800
C8—N2	1.2830 (17)	C17—H19C	0.9800
C8—C9	1.4554 (18)	C18—O7	1.4247 (18)
C8—H8	0.9500	C18—H18A	0.9900
C9—C14	1.4004 (19)	C18—H18B	0.9900
C9—C10	1.4023 (18)	N1—N2	1.3889 (15)
C10—O5	1.3615 (16)	N1—H1'	0.8800
C10—C11	1.3948 (18)	O2—H2	0.8400
C11—C12	1.3869 (19)	O3—H3	0.8400
C11—H11	0.9500	O4—H4	0.8400
C12—O6	1.3672 (16)	O7—H7	0.8400
C12—C13	1.3938 (19)		
			
C6—C1—C2	120.50 (12)	C12—C13—H13	120.4
C6—C1—H1	119.8	C13—C14—C9	121.44 (13)
C2—C1—H1	119.8	C13—C14—H14	119.3
O2—C2—C1	116.85 (12)	C9—C14—H14	119.3
O2—C2—C3	123.07 (11)	O6—C15—H15A	109.5
C1—C2—C3	120.06 (12)	O6—C15—H15B	109.5
O3—C3—C2	125.32 (12)	H15A—C15—H15B	109.5
O3—C3—C4	115.73 (11)	O6—C15—H15C	109.5
C2—C3—C4	118.93 (11)	H15A—C15—H15C	109.5
O4—C4—C5	117.51 (12)	H15B—C15—H15C	109.5
O4—C4—C3	121.43 (12)	O5—C16—H16A	109.5
C5—C4—C3	121.07 (12)	O5—C16—H16B	109.5
C4—C5—C6	119.75 (12)	H16A—C16—H16B	109.5
C4—C5—H5	120.1	O5—C16—H16C	109.5
C6—C5—H5	120.1	H16A—C16—H16C	109.5
C1—C6—C5	119.63 (12)	H16B—C16—H16C	109.5
C1—C6—C7	117.84 (11)	C18—C17—H19A	109.5
C5—C6—C7	122.52 (12)	C18—C17—H19B	109.5
O1—C7—N1	121.44 (12)	H19A—C17—H19B	109.5
O1—C7—C6	121.75 (12)	C18—C17—H19C	109.5
N1—C7—C6	116.79 (12)	H19A—C17—H19C	109.5
N2—C8—C9	120.91 (12)	H19B—C17—H19C	109.5
N2—C8—H8	119.5	O7—C18—C17	111.58 (13)
C9—C8—H8	119.5	O7—C18—H18A	109.3
C14—C9—C10	118.24 (12)	C17—C18—H18A	109.3
C14—C9—C8	121.81 (12)	O7—C18—H18B	109.3
C10—C9—C8	119.95 (12)	C17—C18—H18B	109.3
O5—C10—C11	123.45 (12)	H18A—C18—H18B	108.0
O5—C10—C9	115.45 (11)	C7—N1—N2	119.19 (11)
C11—C10—C9	121.09 (12)	C7—N1—H1'	120.4
C12—C11—C10	118.67 (12)	N2—N1—H1'	120.4
C12—C11—H11	120.7	C8—N2—N1	114.10 (11)
C10—C11—H11	120.7	C2—O2—H2	109.5
O6—C12—C11	123.82 (12)	C3—O3—H3	109.5
O6—C12—C13	114.82 (12)	C4—O4—H4	109.5
C11—C12—C13	121.36 (12)	C10—O5—C16	118.18 (11)
C14—C13—C12	119.17 (13)	C12—O6—C15	118.75 (11)
C14—C13—H13	120.4	C18—O7—H7	109.5

### Hydrogen-bond geometry (Å, °)

**Table:**

*D*—H···*A*	*D*—H	H···*A*	*D*···*A*	*D*—H···*A*
O2—H2···O3	0.84	2.54	2.9325 (13)	109
O3—H3···O2	0.84	2.54	2.9325 (13)	110
O4—H4···O3	0.84	2.25	2.7009 (14)	114
N1—H1'···O7^i^	0.88	2.04	2.8844 (15)	160
O2—H2···O1^ii^	0.84	1.86	2.6871 (13)	170
O2—H2···N2^ii^	0.84	2.57	2.9293 (15)	107
O3—H3···O1^ii^	0.84	1.88	2.7200 (13)	177
O4—H4···O6^iii^	0.84	2.14	2.7366 (14)	127
C14—H14···O2^iv^	0.95	2.42	3.3539 (17)	167

Symmetry codes: (i) *x*, *y*−1, *z*; (ii) *x*+1/2, −*y*+1/2, *z*+1/2; (iii) *x*+1, *y*, *z*+1; (iv) *x*−1/2, −*y*+1/2, *z*−1/2.
